# The fetal phenotype is a trajectory, not a snapshot

**DOI:** 10.1111/aogs.70369

**Published:** 2026-10-27

**Authors:** Florian Recker, Adeline Walter, Ulrich Gembruch

**Affiliations:** ^1^ Department of Obstetrics and Prenatal Medicine University Hospital Bonn Bonn Germany; ^2^ Department of Obstetrics, Faculty of Medicine and University Hospital Cologne University of Cologne Cologne Germany

**Keywords:** fetal phenotype, genetic counseling, human phenotype ontology, prenatal exome sequencing, reporting standards, serial ultrasound, variant reclassification

## Abstract

The fetal phenotype is a trajectory rather than a fixed observation: features emerge, progress, or resolve across gestation, and the anomaly present at 20 weeks is often not the anomaly present at 32. Sequence variants, however, are interpreted against the phenotype supplied when the test is requested, and phenotypic specificity carries evidence weight in variant classification. A scoping review of 59 studies combining serial prenatal phenotyping with sequence‐based testing found that a newly emergent phenotype led to a molecular diagnosis in 16 studies and a variant reclassification in 8; yet a gestational age for every phenotypic observation was reported in 2, a serial imaging protocol in 7, and a reclassification with both direction and evidence code in 1. The clinical signal is real, and the literature cannot quantify it. Five reporting practices are proposed, none of which requires new equipment or funding.

## Graphical abstract

The evolving fetal phenotype. Serial prenatal ultrasound may reveal emerging or changing fetal features that inform reassessment of genetic findings. Reporting the timing of observations, changes in the fetal phenotype and any subsequent revision of variant interpretation helps clarify the contribution of longitudinal imaging to prenatal diagnosis.

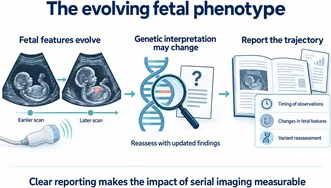

**Key Message**

Serial prenatal imaging demonstrably alters molecular diagnosis and variant interpretation, yet published reports rarely record when features appeared or whether classifications were revisited. The evolving fetal phenotype should be documented and reported in every study of fetal genetic testing.

The mid‐trimester anomaly scan is the point at which fetal medicine commits itself. A structural finding at 20 weeks defines the differential diagnosis, triggers the referral, and—increasingly—determines the phenotype against which a prenatal exome or genome is interpreted.^1^ That phenotype then tends to stay fixed. The report is written, the test is requested, the variant is classified against the features listed on the request form, and the answer comes back. The request form has become, in effect, the clinical description of the fetus.

Prenatal genomic testing is now part of that commitment. Where karyotyping and chromosomal microarray are normal, a fetus with a structural anomaly may be offered a targeted multigene panel, or trio exome or genome sequencing, and in many services the result returns during the pregnancy rather than after it.^2^, ^3^ What comes back is not a simple positive or negative. Under the framework of the American College of Medical Genetics and Genomics and the Association for Molecular Pathology, a variant is placed in one of five classes—pathogenic, likely pathogenic, uncertain significance, likely benign, benign—by combining weighted lines of evidence: how often the variant is seen in population databases, what computational and functional data predict, how it segregates within the family, and how specifically the patient's phenotype matches the disease associated with that gene.^4^ The last of those is the one that matters here. The phenotype is not merely the reason the test was sent; it is one of the inputs to the score. Only pathogenic and likely pathogenic variants support clinical action, and a variant of uncertain significance is by definition one whose evidence does not reach that threshold—so a change in the phenotypic evidence is among the few things that can move it.

The fetus, however, does not hold still. Features emerge, progress, persist, or resolve across the second and third trimesters, and the anomaly present at 20 weeks is frequently not the anomaly present at 32. Routine late‐gestation scanning makes the point quantitatively: A 36‐week scan detects abnormalities not identified earlier in a substantial number of otherwise unremarkable pregnancies,^5^ and the same holds at 35–37 weeks.^6^ What is detected late is not always new. Some of it was present but not resolvable earlier; some of it is the natural history of a monogenic condition unfolding in utero; some of it is what a given gestational age and fetal position permit an operator to see. For the purposes of variant interpretation, the distinction matters less than it appears to, because the consequence is identical in all three cases: the phenotype available in the third trimester is not the phenotype that was submitted in the second.

This has a specific consequence for the laboratory that is rarely made explicit. Because phenotypic specificity carries evidence weight—most directly through PP4, the criterion for a phenotype highly specific to a single gene, but in practice throughout the reasoning that decides whether a variant of uncertain significance is reported, held, or resolved—a classification is a function of the phenotype supplied at the moment of classification.^4^ If the phenotype moves, the classification that was correct in the second trimester may no longer be the best‐supported classification in the third. Whether laboratories and fetal medicine services systematically revisit that question is not something the field has established, and—as set out below—the published literature does not currently allow it to be established.

The two cohorts that brought prenatal exome sequencing into practice both said as much, in their own limitations. PAGE noted that phenotypic information available prenatally was incomplete relative to what emerged later^2^; the Columbia cohort made a structurally identical observation.^3^ A subsequent cohort study of progressive prenatal sequencing pathways reported evolving phenotypes as a feature of the pathway rather than an artifact of it,^7^ and a direct comparison of prenatal with postnatal exome sequencing found diagnoses that were missed prenatally precisely because the phenotype was not yet informative.^8^ These are not marginal observations from small series. They are the limitation sections of the studies on which current practice rests.

## 1 HOW THE STUDIES WERE IDENTIFIED

PubMed and Europe PMC were searched from inception to 5 August 2026; Embase, Web of Science, Scopus, and the Cochrane Library were not accessible, and that limitation is reported in full in the review. Of 4364 unique records screened, 190 were adjudicated individually against the full record text and 131 of those were excluded, most often because the phenotype was documented at a single timepoint. Fifty‐nine studies were eligible: Each described a human fetal phenotype documented on at least two occasions, or compared prenatally with postnatally, together with multigene panel, exome, or genome sequencing and at least one diagnostic, interpretive, or counseling outcome. Reports based on karyotype or chromosomal microarray alone were excluded. Each included study was then scored against eight reporting items chosen for this question, taken from the text of the report itself.

Study design was deliberately not used as a restriction, and the resulting mix—23 case reports, 19 cohort studies, 13 case series, and 4 other designs—needs a word of explanation, since the instinct to exclude single cases is a sound one in most contexts. The question was whether a documented change in fetal phenotype altered the interpretation of a sequence variant in a given pregnancy. That event, where it is described at all, is usually described as a case report. Restricting to cohorts would have removed most of the documented instances without making the frequency of the event any more calculable and would have concealed the very distribution the review set out to map. Case reports were therefore counted as instances of documentation and never pooled into a rate: no meta‐analysis was performed, and single‐study proportions are reported with their numerator, denominator, and source named.

## 2 WHAT THE LITERATURE CAN AND CANNOT SHOW

A recent scoping review of this question by the present authors charted 59 studies that combined serial or repeated prenatal phenotyping with sequence‐based genetic testing and at least one diagnostic, interpretive, or counseling outcome. Two findings are worth putting in front of this readership, and the figure sets them side by side.

The clinical signal is unambiguous at the level of the individual pregnancy (Figure 1A). A newly emergent phenotype led to a molecular diagnosis in 16 of 59 studies (27.1%). A variant reclassification, or a classification explicitly described as phenotype‐dependent, was documented in 8 studies (13.6%). Twelve studies (20.3%) reported a refined phenotype without any documented change in variant classification, and 3 (5.1%) reported that serial imaging changed a testing or surveillance decision without changing a classification. One study reported an explicitly negative result and deserves emphasis disproportionate to its single‐study weight: in 203 fetuses with renal agenesis, five children with postnatally identified phenotypes had undergone prenatal trio exome sequencing, and reanalysis incorporating the new postnatal features remained negative in all five.^9^ New phenotype does not reliably buy diagnostic yield. Any framework built on the assumption that it does—including the one proposed below—has to accommodate that result.

**FIGURE 1:**
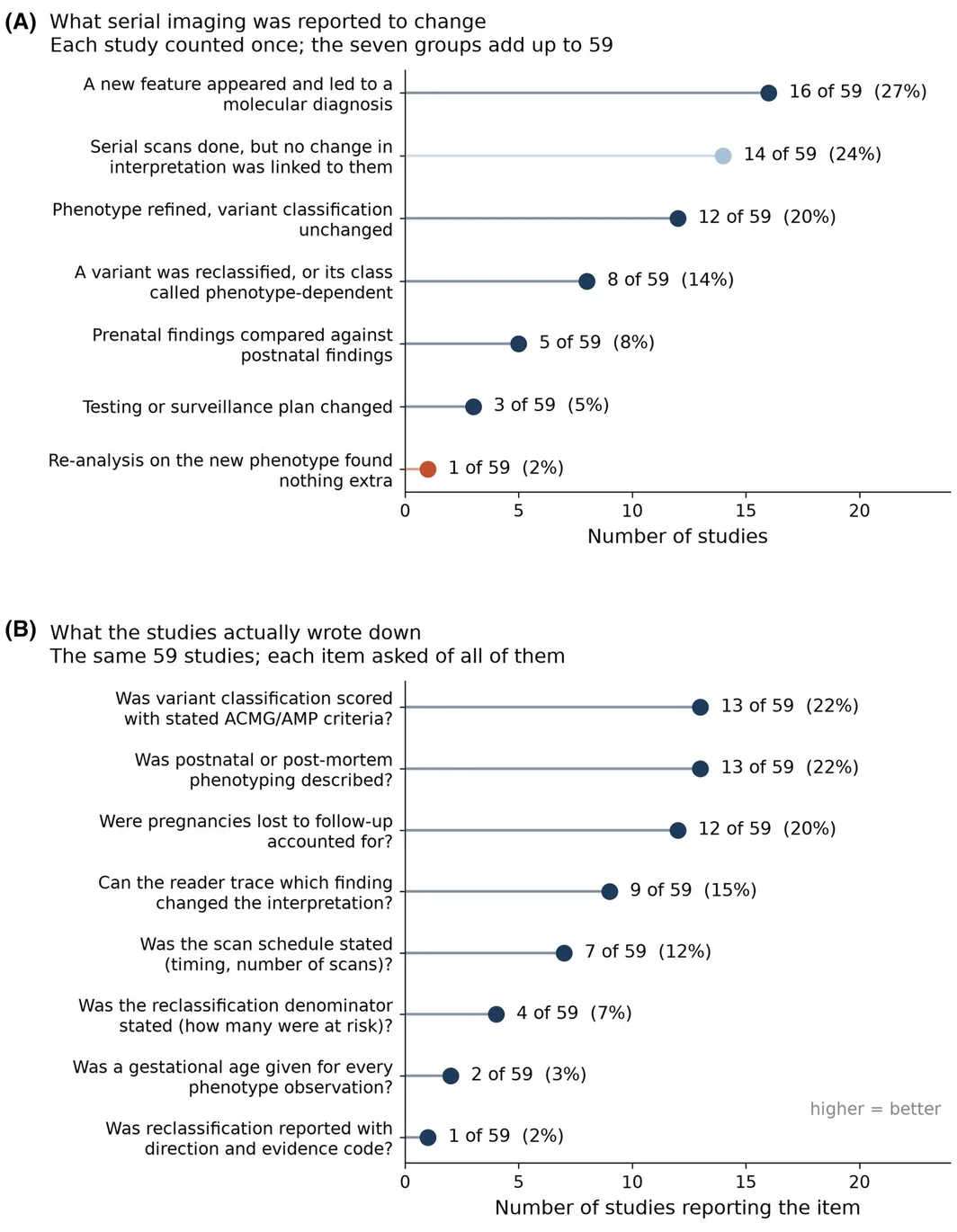
What serial prenatal imaging was reported to change, and what the reports wrote down (59 studies). Both panels describe the same 59 studies and both are counted the same way, as a number out of 59. (A) What each study reported serial imaging to have changed. Every study is placed in one group only, so the seven groups add up to 59. The pale blue group is the studies that reported serial imaging but linked no change in interpretation to it; the red group is the single study that re‐analyzed sequencing data against a new phenotype and gained no additional diagnosis. In panel (B), ACMG/AMP denotes the classification criteria of the American College of Medical Genetics and Genomics and the Association for Molecular Pathology. (B) How many of the same 59 studies reported each of eight items that a reader needs before results from different studies can be added together. In short: Panel (A) is what was found, panel b is what was written down.

The second finding is the one that deserves the reader's attention (Figure 1B). Across those same 59 studies, the gestational age at *every* phenotypic observation was reported in 2 (3.4%). A serial imaging protocol—how many scans, at what intervals—was stated in 7 (11.9%). A reclassification event reported with both its direction and the evidence code that changed appeared in exactly one study (1.7%). Restricting to the 17 studies charted from full text rather than abstract (not plotted) raises these rates but does not repair them: Gestational age at every observation reaches 11.8%, the serial protocol 23.5%, and directional reclassification with an evidence code remains a single study. The deficit is not an artifact of abstract‐only charting, and it is not a judgment on the conduct of the studies. It is a statement about what their reports contain.

The consequence is that the field cannot currently count its own key events. The review could not estimate how often a fetal phenotype changes in a way that alters interpretation, at what gestational age this most often happens, or how often reassessment changes management—not because the studies are poor, but because the quantities are not in the text. A further 14 studies (23.7%) reported serial imaging alongside sequencing but attributed no interpretive change to the phenotype change; these were recorded as unattributed rather than negative because in most cases the source authors simply did not address the question, and scoring silence as a null result would be an error.

## 3 WHY THIS IS A REPORTING PROBLEM, NOT A TECHNOLOGY PROBLEM

The composition of the evidence base is itself informative. Of the 59 studies, 23 were case reports and 13 case series. A literature dominated by single cases is what a field produces when it documents striking trajectories it happens to notice, rather than measuring trajectories it set out to observe. The imaging is already being performed—these pregnancies were re‐scanned, often repeatedly, as a matter of routine care. What is missing is the convention that turns a second look into an aggregable observation.

Two clinical pictures familiar to this readership illustrate what is at stake. In fetal cardiomyopathy, the median gestational age at diagnosis falls in the late second or third trimester, ventricular function and hypertrophy evolve after the mid‐trimester examination, and hydrops develops later in gestation in a substantial minority.^10^, ^11^ In hydrometrocolpos, the fetus may be unremarkable at 20 weeks and present later with a progressively distending pelvic mass whose differential shifts as it enlarges.^12^, ^13^ In both, the phenotype that would most inform variant interpretation is not the phenotype that was available when the test was sent—and in both, a laboratory working from the 20‐week request form is reasoning from an out‐of‐date description.

None of this will be news in every center. Units exist in which fetal features are already coded to ontology terms at the time of the scan, re‐imaging intervals are protocolized, and a variant of uncertain significance is formally revisited when the phenotype moves; for those services the practices set out below will read as a description of ordinary work. The deficit is not that the work is not being done. It is that where it is done, it does not reach the literature in a form anyone else can count. A reassessment that takes place in a multidisciplinary meeting and is recorded in a hospital system, but not in the published report of the cohort, is invisible to every subsequent reader—and so cannot inform a guideline, a service specification, or the next review of this question.

## 4 FIVE PRACTICES, NONE OF WHICH REQUIRES NEW EQUIPMENT OR FUNDING

Record a gestational age with every phenotypic observation, not only with the first. This single item was reported in 2 of 59 studies, and without it no analysis of *when* phenotypes change is possible.

Code fetal features to Human Phenotype Ontology terms at the time of the scan,^14^ so that an index phenotype and a later phenotype are machine‐comparable rather than narratively comparable.

State explicitly which features were sought and not found. An absent feature at 32 weeks is evidence; an unrecorded absence is not, and the two are indistinguishable to any later reader.

When a variant is reassessed, date the reassessment and name the evidence code that changed.^4^ A classification history is a different object from a classification, and only the former can be audited.

State the denominator: How many fetuses were re‐imaged, how many phenotypes changed, how many classifications were revisited as a result. In that review the denominator for reclassification was stated in half of the studies in which reclassification was at issue.

A variant of uncertain significance reported at 22 weeks should not be treated as a closed question at 32. Where a fetus is re‐imaged and the phenotype has moved, the laboratory should be told, and the reassessment documented with the same rigor as the original report. This is also, and perhaps mainly, a request to authors, reviewers, and editors. A report of prenatal sequencing in fetuses with structural anomalies could reasonably be expected to state when each phenotypic feature was observed, how many fetuses were re‐imaged, and whether any classification was revisited as a result—three items that cost a sentence each, and that no current reporting standard for this literature requires. None of this is a call for new trials or new machines. It is a call to write down what is already seen—and it is what would allow the next review of this question to report an effect size rather than a reporting deficit.

## AUTHOR CONTRIBUTIONS

Florian Recker conceived this Commentary, designed and conducted the scoping review on which it draws, analyzed the charted data, produced Figure 1 and drafted the manuscript. Adeline Walter contributed to the clinical framing of the argument, to the interpretation of the fetal cardiomyopathy and hydrometrocolpos material, and to critical revision of the manuscript. Ulrich Gembruch contributed to the conception of the argument, provided senior clinical oversight of the fetal medicine content, and critically revised the manuscript. All three authors approved the submitted version and accept accountability for its content (draft attribution—to be amended to reflect the actual division of work).

## FUNDING INFORMATION

This work received no specific funding.

## CONFLICT OF INTEREST STATEMENT

The authors report no conflicts of interest.

## Data Availability

The data that support the findings of this study are available from the corresponding author upon reasonable request.
